# Cell lysis-free quantum dot multicolor cellular imaging-based mechanism study for TNF-α-induced insulin resistance

**DOI:** 10.1186/s12951-015-0064-x

**Published:** 2015-01-27

**Authors:** Min Jung Kim, Sabarinathan Rangasamy, Yumi Shim, Joon Myong Song

**Affiliations:** College of Pharmacy, Seoul National University, Seoul, 151-742 South Korea

**Keywords:** Multicolor cellular imaging, Insulin resistance, Quantum dot, Inflammation

## Abstract

**Background:**

TNF-α is an inflammatory cytokine that plays an important role in insulin resistance observed in obesity and chronic inflammation. Many cellular components involved in insulin signaling cascade are known to be inhibited by TNF-α. Insulin receptor substrate (IRS)-1 is one of the major targets in TNF-α-induced insulin resistance. The serine phosphorylation of IRS-1 enables the inhibition of insulin signaling. Until now, many studies have been conducted to investigate the mechanism of TNF-α-induced insulin resistance based on Western blot. Intracellular protein kinase crosstalk is commonly encountered in inflammation-associated insulin resistance. The crosstalk among the signaling molecules obscures the precise role of kinases in insulin resistance. We have developed a cell lysis-free quantum dots (QDots) multicolor cellular imaging to identify the biochemical role of multiple kinases (p38, JNK, IKKβ, IRS1^ser^, IRS1^tyr^, GSK3β, and FOXO1) in inflammation-associated insulin resistance pathway with a single assay in one run. QDot-antibody conjugates were used as nanoprobes to simultaneously monitor the activation/deactivation of the above seven intracellular kinases in HepG2 cells. The effect of the test compounds on the suppression of TNF-α-induced insulin resistance was validated through kinase monitoring. Aspirin, indomethacin, cinnamic acid, and amygdalin were tested.

**Results:**

Through the measurement of the glycogen level in HepG2 cell treated with TNF-α, it was found that aspirin and indomethacin increased glycogen levels by almost two-fold compared to amygdalin and cinnamic acid. The glucose production assay proved that cinnamic acid was much more efficient in suppressing glucose production, compared with MAP kinase inhibitors and non-steroidal anti-inflammatory drugs. QDot multicolor cellular imaging demonstrated that amygdalin and cinnamic acid selectively acted via the JNK1-dependent pathway to suppress the inflammation-induced insulin resistance and improve insulin sensitivity.

**Conclusion:**

The regulatory function of multiple kinases could be monitored concurrently at the cellular level. The developed cellular imaging assay provides a unique platform for the understanding of inflammation and insulin resistance signaling pathways in type II diabetes mellitus and how they regulate each other. The results showed that amygdalin and cinnamic acid inhibit serine phosphorylation of IRS-1 through targeting JNK serine kinase and enhance insulin sensitivity.

## Background

The worldwide prevalence of diabetes has progressively risen over the past 30 years. According to the World Health Organization, the global number of people with diabetes is 371 million; of this population, around 90% suffer from type II diabetes [1]. Cardiovascular disease, obesity, kidney failure, diabetic retinopathy, and neuropathy are associated with type II diabetes mellitus (T2DM). Inflammation and insulin resistance play a vital role in the above T2DM-related diseases.

Perturbations in downstream proteins involved in insulin signaling pathways have been found in insulin resistance and inflammation-associated T2DM. Hundreds of protein molecules are intricately involved in the insulin signaling pathway. The insulin receptor substrate (IRS1-4) proteins, phosphatidylinositol 3 kinase (PI3K), and AKT/protein kinase B (PKB) are considered as the best junctions for potential crosstalk with other pathways [2]. IRS protein undergoes serine phosphorylation in response to such stimuli as TNF-α and negatively controls IRS signaling. In insulin-resistant states, the phosphorylation of IRS1 is elevated, and it activates many downstream kinases, such as extracellular signal-regulated kinase ERK, S6 kinase, and c-Jun-N-terminal kinase. TNF-α also functions through its receptor on the cell surface and targets multiple components, including the insulin receptor, the insulin receptor substrate (IRS), glucose synthesis through GSK3, gluconeogenesis via FOXO1, and glucose transporter 4 (Glut4) in insulin-signal transduction [3]. On the other hand, TNF-α is considered as one of the molecular targets for suppressing inflammation in insulin-resistant conditions. TNF-α induces the TAK kinase-IKKβ/p38/JNK kinase axis and finally promotes PPAR γ inhibition [4]. Common protein kinases located in the signaling cascade are responsible for crosstalk. In the case of inflammation-associated insulin resistance, there are several instances of crosstalk between insulin signaling composed of IRS1-4 proteins, PI3K, AKT/protein kinase B (PKB), and mitogen-activated protein kinases (MAPKs, i.e., p38, JNK, and IKKβ) of inflammatory signaling. The crosstalk intercommunication between the inflammation and insulin-resistant kinases complicates our understanding of signal propagation. Crosstalk creates a barrier to deciphering the biochemical function of individual protein kinases in metabolic disturbances. Consequently, inflammation-associated T2DM disease mechanisms are poorly understood. One of the most daunting challenges is to identify the function of individual signaling molecules in the presence of two disease conditions, such as inflammation and insulin resistance.

Until now, Western blot has been used as the main tool to monitor signaling transductions which lead to insulin resistance. The current approaches to evaluate signaling molecule activity are deficient in terms of sensing phosphorylated proteins, tracing the junctions of crosstalk, sensing variation in intensity level, and detecting morphological changes in cells, specifically to identify individual protein molecules in a cell population. Moreover, the current approaches are qualitative in nature. The quantification of the amount of specific proteins can be difficult. Pure acquisition of a particular protein from a pool of various proteins is not easy. Antibodies may exhibit some off-target binding, which can cause poor results. Performing a Western blot properly with good results can be challenging, and requires a well-trained staff. Moreover, during the entire procedure, the temperature and pH must be maintained properly. Loss of protein during the preparation of samples through many experimental steps, including cell lysis, is also possible. As a result, large numbers of cells are needed due to the degree of cell lysis. In spite of the large number of cells to be lysed, insensitivity of Western blot makes it difficult to correctly define activation or deactivation of the relevant proteins. In this work, cell lysis-free multicolor cellular imaging-based mechanism study for TNF-α-induced insulin resistance is introduced. Proteins involved in inflammation and insulin signaling were concurrently monitored without cell lysis. This approach is based on the use of quantum dot-antibody conjugates as nanoprobes for simultaneously observing relevant proteins. The nanoprobe can penetrate into the cellular membrane without cell lysis and bind to the target protein via its antibody. Several antibodies are capable of being attached to the surface of quantum dot by covalently bonding to 4-(maleimidomethyl)-1-cyclohexanecarboxylic acid N-hydroxysuccinimide ester (SMCC) compound. QDdots have superior optical properties, such as photostability, a high level of brightness, and a higher photobleaching threshold [5] than that of the organic dyes used in classic immunofluorescence. In fact, QDdots are 10–20 times brighter and have several thousand times more photobleaching resistance than organic dyes [6]. Additionally, QDots can be excited simultaneously at a particular excitation wavelength compared to organic dyes [7]. This property enables multicolor cellular imaging for simultaneous monitoring of many relevant proteins involved in TNF-α-induced insulin resistance. Biofunctionalized QDots have been actively used as optical probes for cell imaging. CdSe/ZnS QDots modified with dentate-like alkyl chains and multiple carboxyl groups were developed and conjugated with BRCAA1 and Her2 antibody. The QDots were used for *in vitro* MGC803 cell labeling and *in vivo* targeted imaging of gastric cancer cells [8]. A hydrophilic semiconductor quantum dot-peptide forster resonance energy transfer nanosensor was fabricated to monitor the activity of kallikrein, a key proteolytic enzyme functioning at the initiation of the blood clotting cascade [9]. Avian influenza H5N1 pseudotype virus (H5N1p) was labeled with NIR-emitting QDots by bioorthogonal chemistry. The prepared QDot-H5N1ps were used to visualize respiratory viral infections in mouse lung tissue in real-time [10]. QDot-tagged photonic crystal beads were successfully applied to the multiplex immunoassay of tumor markers [11]. Compared to Western blot, the present method consumes a much smaller number of cells because of the direct monitoring of proteins in the cytosol without cell lysis. First of all, direct monitoring of target proteins without lysis definitely increases the accuracy of the validations regarding the efficacy of test compounds for suppression of inflammatory signaling and enhancement of insulin signaling. High intensity, as well as lack of protein loss, leads to enhancement of accuracy level. As a result, the readouts are a closer reflection of physiological intracellular protein expression. Moreover, multicolor cellular imaging is more similar to *in vivo* results than biochemical assays, resulting in reducing failures in clinical trials. The entire procedure can be carried out faster than Western blot. In addition, more than one protein is easily monitored through a set of samples simultaneously. We have undertaken a quantitative approach and computational methodology to identify the components of two signaling pathways at the same time. Multicolor cellular imaging acts as a catalyst for the rational targeting of specific kinases, mainly focusing on their functional role in disease mechanisms. This assay can be considered as a fundamental tool for concurrently defining the biochemical functions of multiple kinases in multiple signaling pathways with a single assay in one run.

Herein, we propose a new set of multicolor cellular imaging to study biochemical cell-signaling networks which are convoluted, and contain different points of regulation, signal divergence, and crosstalk with other transduction pathways. Amygdalin and cinnamic acid were examined to elucidate their molecular mechanism on the suppression of TNF-α-induced insulin resistance using multicolor cellular imaging based on QDot nanoprobe. Seven kinases were monitored in HepG2 cells treated with TNF-α for the concurrent monitoring of inflammatory and insulin signaling. Serine kinases, such as JNK, IKK, and p38α were observed to verify their roles on serine phosphorylation of IRS-1. Furthermore, GSK3 and FOXO1 were monitored as target proteins for the enhancement of glycogen synthesis and suppression of gluconeogenesis induced by amygdalin and cinnamic acid.

## Results and discussion

**Figure 1:**
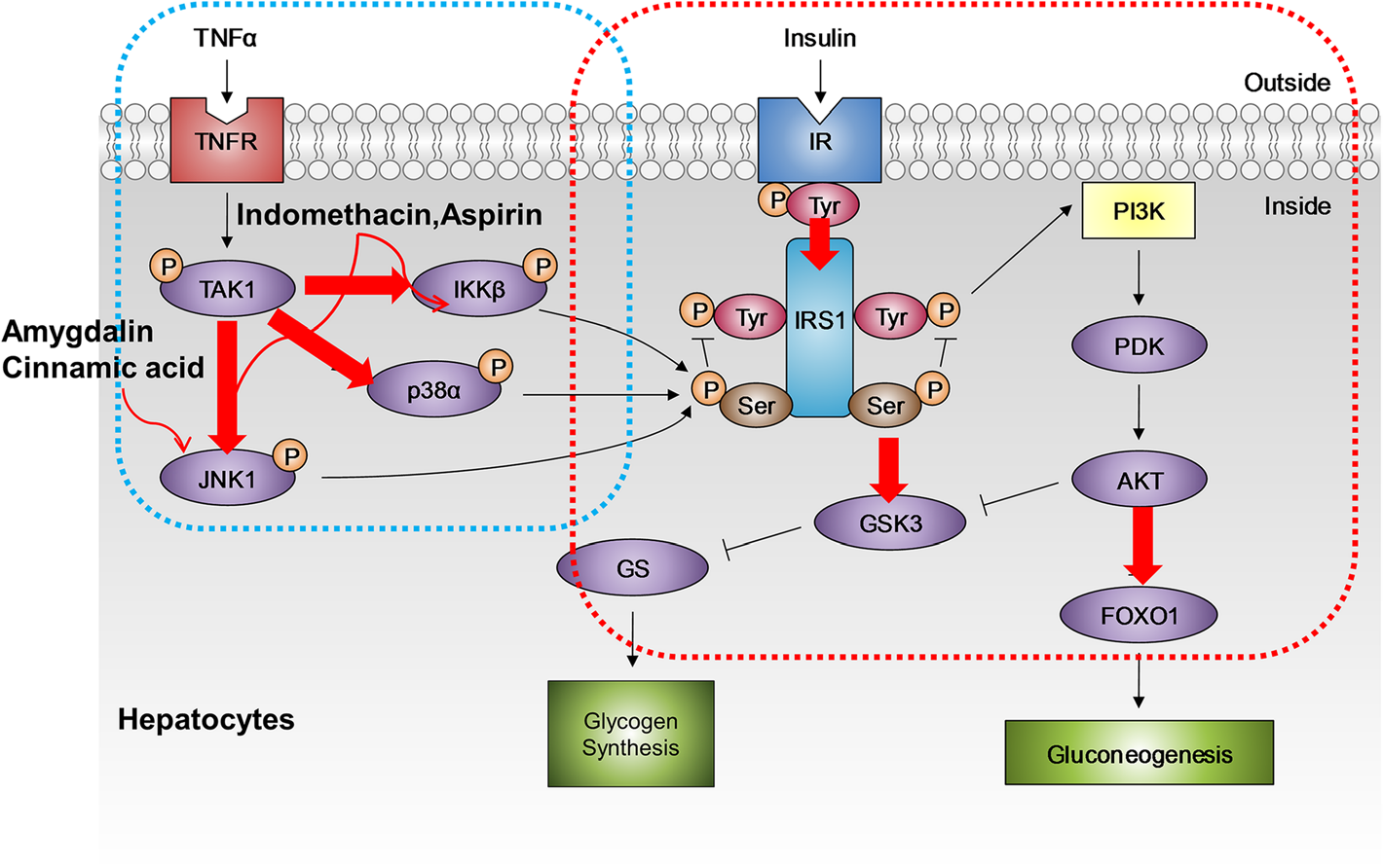
**Effect of four different chemicals on inflammation-associated insulin signaling pathway in HepG2 cells.** The QDot HCS assay was developed for various key kinases involved in inflammation-associated IR that are indicated with a red arrow and validated by specific inhibitors and anti-inflammatory drugs as shown. The p38α, JNK1, and IKKβ involved in the inflammatory pathway and IRS1^ser307^, IRS1^tyr^, FOXO1, and GSK3β associated with the insulin signaling pathway were simultaneously monitored by QDot multicolor cellular imaging.

**Figure 2:**
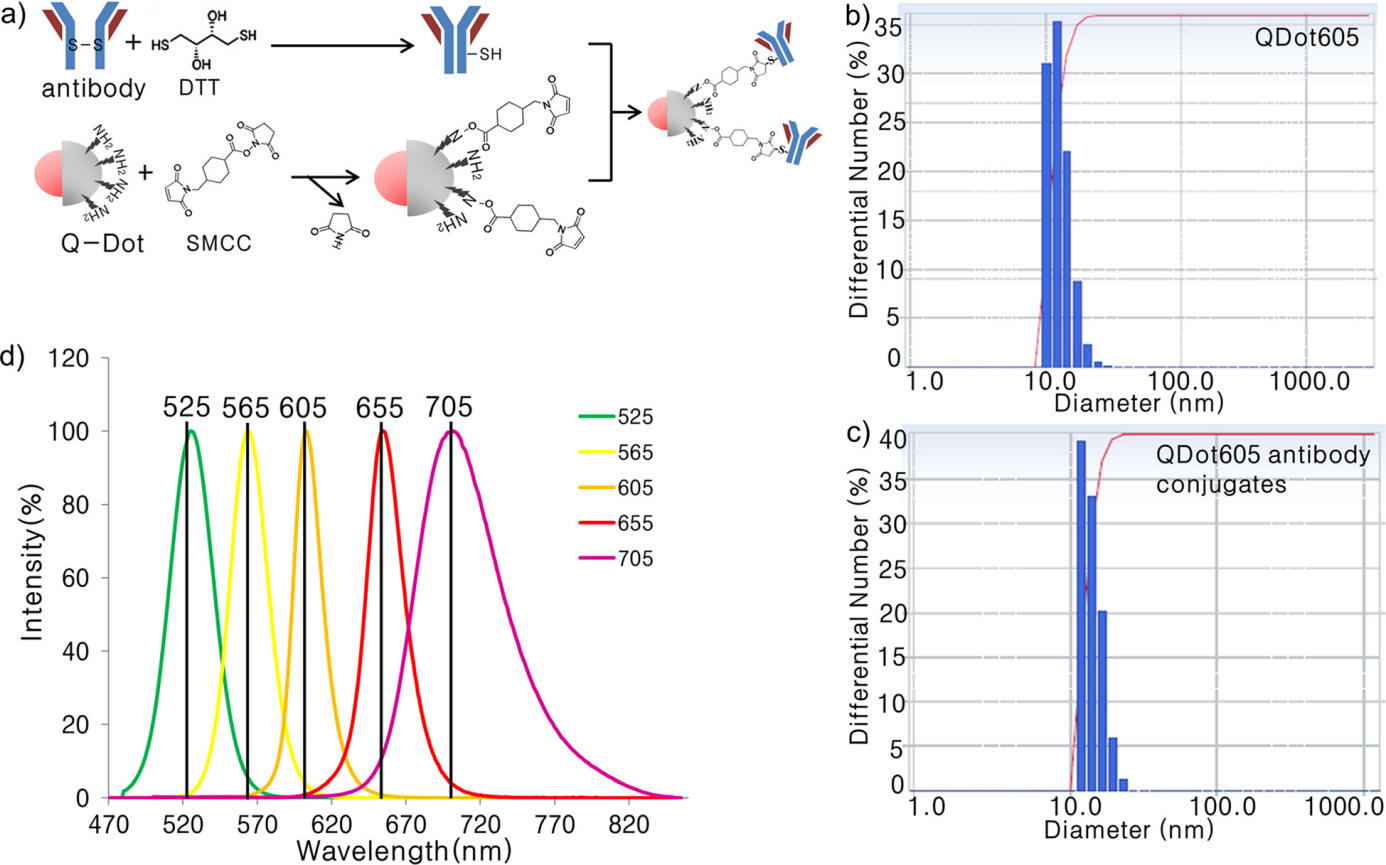
**Characterization of QDot-antibody conjugates. (a)**. A schematic diagram of QDot-antibody conjugation mediated with SMCC. **(b,c)**. Particle size analysis of QDot605 and QDot605-antibody conjugate obtained through dynamic light scattering. Particle size measurements were executed using Photal ELSZ-1000 instrument (Otsuka Electronics Ltd., Osaka, Japan). Before the particle size measurements, QDot605 and QDot605-antibody conjugates were diluted with 0.22 μm filtered water. Average diameter calculated was 11.12 nm for QDot605 and 20.4 nm for QDot605-antibody conjugate by particle size analysis. **(d)**. Emission spectra of QDot525, QDot565, QDot 605, QDot655, and QDot705.

**Figure 3:**
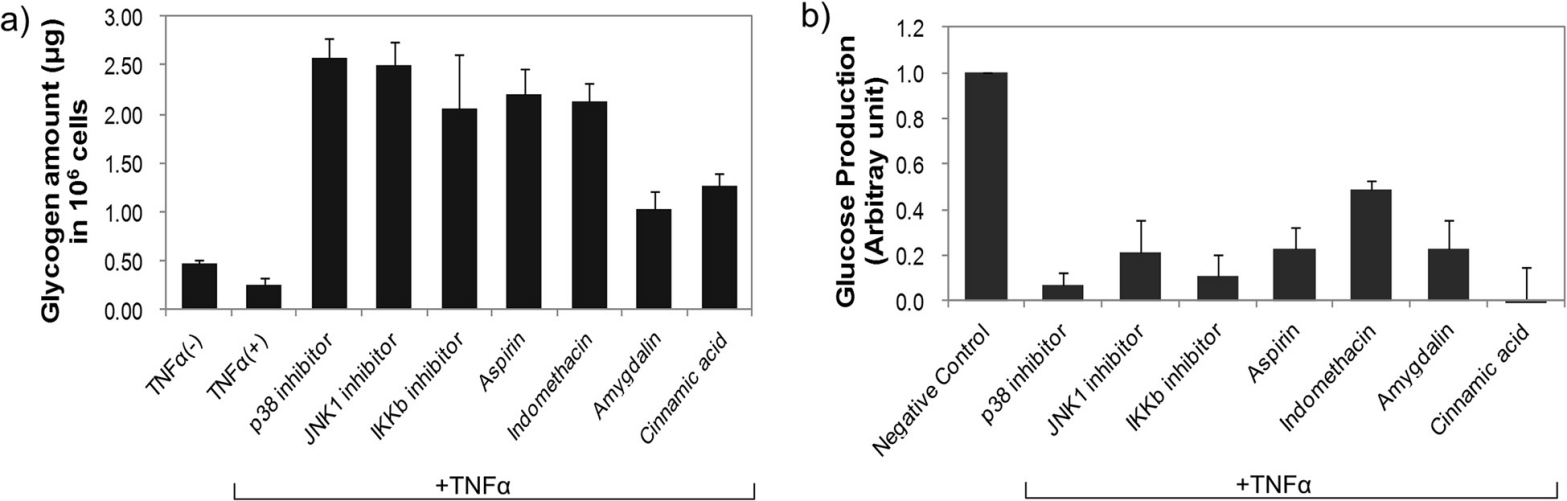
**Quantification of glycogen and glucose production in HepG2 hepatocytes. (a)**. Enhancement of glycogen content in inhibitor-pretreated HepG2 hepatocytes in the presence of TNF-α. Human HepG2 hepatocytes were plated in serum-free DMEM media for 24 h prior to testing. Inhibitors were added 30 min before stimulation with 10 ng/ml TNF-α. After 5 h, the cells were treated with 100 nM insulin for 10 min, and further steps were carried out according to the manufacturer’s instructions (Biovision, Milpitas, CA, USA). All values are mean ± SD, n = 3. **(b)**. The effect of MAP kinase inhibitors on glucose production. All values are mean ± SD, n = 3.

Figure 1 shows a schematic model for TNF-α-induced insulin resistance. TNF-α is the most extensively studied cytokine from the point of view of insulin resistance. TNF-α acts by direct and indirect mechanisms to converge at multiple signaling nodes in the way of insulin action. Direct mechanisms include the stimulation of the serine phosphorylation of IRS-1 and decreasing the expression of IRS-1 and GLUT-4 [12]. Indirectly, TNF-α also interacts with muscle cells, liver cells, and adipose tissue to evoke insulin resistance. These stimuli may activate overlapping signal pathways via common upstream kinases in the insulin pathway, such as IRS1-4 proteins, PI3K and AKT/protein kinase B (PKB), and the activation of several kinases, such as p38α, JNK1, and IKKβ in inflammatory signaling. The crosstalk between inflammation and insulin signaling is a major hurdle for understanding the molecular link between the two pathways. The present approaches to studying cellular signaling mechanisms suffer from fluctuation in the activities of intracellular kinases and reproducible results. Therefore, we developed a novel QDot-based quantitative multicolor cellular imaging for simultaneous analysis and monitoring of the activity of various kinases in both disease mechanisms. As shown in Figure 2(a), the phospho-p38α, phospho-JNK1, phospho-IKKβ, phospho-IRS1^ser307^, phospho-IRS1^tyr^, phospho-GSK3β, and phospho-FOXO1 antibodies were conjugated with 565, 565, 565, 605, 705, 525, and 655 QDots, respectively, using an antibody conjugation kit for deciphering the insulin resistance pathway. The conjugate between QDot and antibody was verified through a dynamic light scattering and zeta potential measurement. Figure 2(b) and (c) show particle sizes of QDot605 and QDot605-antibody conjugate measured with dynamic light scattering. As a result of conjugation, the size of QDot605-antibody conjugate increased compared to QDot605. Table 1 summarizes particle sizes and zeta potential values of QDots and QDot-antibody conjugates used in this study. Antibodies conjugated to QDots have different structures and charges. As a result, the size of QDot-antibody conjugate was not proportional linearly to that of QDot. Zeta potential values of QDot-antibody conjugates were larger than those of QDots due to the conjugation between QDot and antibody. Zeta potential values of QDot-antibody conjugates were hardly changed three weeks after the conjugation. Emission spectra of five different QDots are very narrow (Figure 2[d]), which is appropriate for the elimination of crosstalk in high-content cell-based assay. In addition, acousto-optic tunable filter (AOTF) can contribute greatly to the spectral overlap-free high-content monitoring. AOTF allows cellular imaging at particular single wavelengths devoid of spectral overlap among QDot-antibody conjugates. The crosstalk-free high-content monitoring of inflammation and insulin signal transduction was attempted using QDot-antibody conjugates and AOTF in this work. We investigated the effect of various inflammatory kinase inhibitors acting on insulin signaling and evaluated the compound efficacies for the functional ability to inhibit gluconeogenesis and enhance glycogen content, with the goal of identifying potential kinase for suppressing inflammation-induced insulin resistance. Our experiment also addresses the suitability of multicolor cellular imaging for studying kinase crosstalk operating in complex diseases, such as inflammation-associated insulin resistance, at a single shot in a single cell. Surprisingly, amygdalin and cinnamic acid acted selectively via the JNK1-dependent pathway to improve insulin sensitivity. The HepG2 cell line was chosen for this study as it is a perpetual, epithelial cell line derived from well-differentiated hepatocellular carcinoma. These cells solely depend upon exogenous growth factors for survival and respond to stimuli, such as TNF-α, adiponectin, leptin, etc. To study the causal role of elevated cytokines levels and MAP kinase inhibitors in hepatic insulin resistance, HepG2 cells were treated with inhibitors 30 min prior to stimulation with 10 ng/ml TNF-α for 5 h. The cells were then treated with 100 nM insulin for 10 min. Past studies using the obese TNF-α^+/+^ mice model have shown disturbances in insulin signaling in response to TNF-α [13]. Therapies that have successfully targeted TNF-α, including genetic silencing of the TNF-α or TNF receptor in mice, have demonstrated significant efficacy in the treatment of obesity-related insulin resistance. Therefore, we established the insulin resistance model *in vitro* using TNF-α stimulation of HepG2 cells. The glycogen amount decreased in the TNF-α treated group, suggesting compromised insulin sensitivity. The MAP kinase inhibitors suppressed the effect of TNF-α on the insulin transduction pathway and consequently resulted in elevated levels of glycogen content (Figure 3a). The inflammatory signaling inhibitors, such as p38α kinase inhibitors (SB203580), JNK1 inhibitors (SP600125), IKKβ inhibitors, aspirin, and indomethacin increased glycogen levels by almost two-fold in comparison to amygdalin and cinnamic acid. Glycogen content in HepG2 cells treated with p38, JNK1, IKKβ inhibitors, aspirin, and indomethacin were four to five times higher than that of the control; whereas, glycogen content in amygdalin or cinnamic acid-treated HepG2 cells were two to three times higher than that of the control [14]. As shown by a glucose production assay, exposure of HepG2 cells to inhibitors reduced gluconeogenesis in comparison to the control group (Figure 3b). Cinnamic acid was much more potent in suppressing glucose production than MAP kinase inhibitors and non-steroidal anti-inflammatory drugs. The rate of hepatic glucose production by amygdalin was similar to that of aspirin.

**Table 1:** **Summary of particle sizes and zeta potential values of QDots and QDot-antibody conjugates**

**Sample**	**Particle size (nm)**	**Zeta potential (mV)**
**QDot525**	9.1	−2.1
**QDot525 antibody conjugate**	15.16	−13.5
**QDot565**	10.3	−2.4
**QDot565 antibody conjugate**	20.3	−11.98
**QDot605**	13.7	−3.72
**QDot605 antibody conjugate**	16.1	−16.65
**QDot655**	15.9	−4.5
**QDot655 antibody conjugate**	20.4	−17.6
**QDot705**	17.1	−5.54
**QDot705 antibody conjugate**	23.2	−18.7

**Figure 4:**
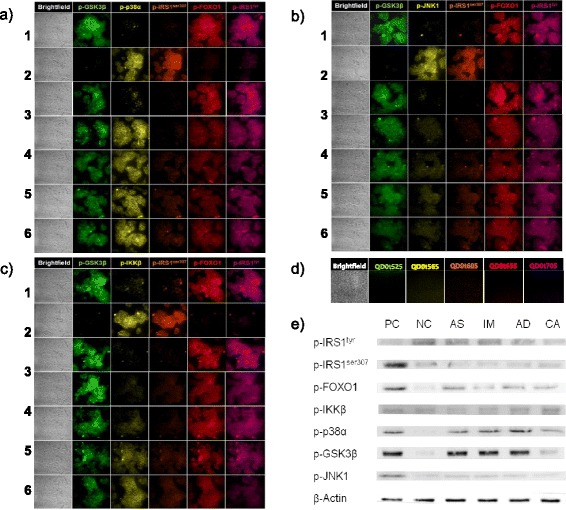
**High-content cellular images of kinases. (a,b,c)**. Multicolor cellular imaging showing simultaneous monitoring of the upregulation/downregulation of five kinases in HepG2 cells. Five kinases: p-GSK3β, p-p38α, p-IRS1^ser307^, p-FOXO1, and p-IRS1^tyr^. **(a)** The positive control group was stimulated with TNF-α; whereas, in the negative control, TNF-α treatment was absent. For inhibitor studies, HepG2 cells were plated in serum-free DMEM media for 24 h prior to testing. P38 inhibitor was added 30 min before stimulation with 10 ng/ml TNF-α (for 5 h), and the cells were treated with 100 nM insulin. The cells were treated with formaldehyde for fixing the cells following permeability enhancement using saponin. The QDot-antibody conjugate (p-p38α, p-JNK1, p-IKKβ, p-IRS1^ser^, p-IRS1^tyr^, p-GSK3β, and p-FOXO1) treatment was done. Quantitative estimation and monitoring of the phosphorylation of the five kinases were simultaneously carried out according to emission wavelengths mentioned in the experimental section. 1: Positive Control, 2: Negative Control, 3: p38 inhibitor (SB203580), 4: Aspirin treatment, 5: Indomethacin treatment, 6: Amygdalin treatment, 7: Cinnamic acid treatment. **(b)** 1: Positive Control, 2: Negative Control, 3: JNK-1 inhibitor (SP600125), 4: Aspirin treatment, 5: Indomethacin treatment, 6: Amygdalin treatment, 7: Cinnamic acid treatment. **(c)** 1: Positive Control, 2: Negative Control, 3: IKKβ inhibitor, 4: Aspirin treatment, 5: Indomethacin treatment, 6: Amygdalin treatment, 7: Cinnamic acid treatment. **(d)** Cellular images of HepG2 cells treated with non-conjugated QDots (QDot525, QDot565, QDot605, QDot655, QDot705). **(e)** Western blot analysis of upregulation/downregulation of seven different kinases in HepG2 cells. TNF-α induction was absent in the negative control (NC). The positive control (PC) was stimulated with TNF-α. AS: Aspirin, IM: Indomethacin, AD: Amygdalin, CA: Cinnamic acid.

Crosstalk among the common signaling molecules complicates our understanding of the relation between inflammation and insulin resistance. Our next goal was to develop novel QDot-based multicolor cellular imaging for simultaneous monitoring of inflammatory and insulin signaling pathway kinases. For the first time, the QDot immunoassay was developed for understanding of TNF-α-induced insulin resistance using the quantitative multicolor cellular imaging. The insulin signaling and one of three inflammatory MAP kinases (p38α, JNK1, and IKKβ) were simultaneously observed. Selective binding of QDot-antibody conjugates to targeted proteins is essential for the successful observation of intracellular signal transduction related to TNF-α-induced insulin resistance. This novel assay is quite useful in tracing kinases without morphological changes in the cell being investigated. The inflammatory pathways via MAP kinases (p38α, JNK1, and IKKβ) were not activated in the negative control group free of any TNF-α stimulation, as shown in Figure 4. In the positive control group, the HepG2 cells were treated with TNF-α to mimic the inflammation-induced insulin-resistant conditions. TNF-α stimulated the phosphorylation of MAP kinases (p38α, JNK1, and IKKβ) and IRS1^ser307^, and inhibited insulin downstream kinases (GSK3β, FOXO1, and IRS1^tyr^) (Figure 4).

**Figure 5:**
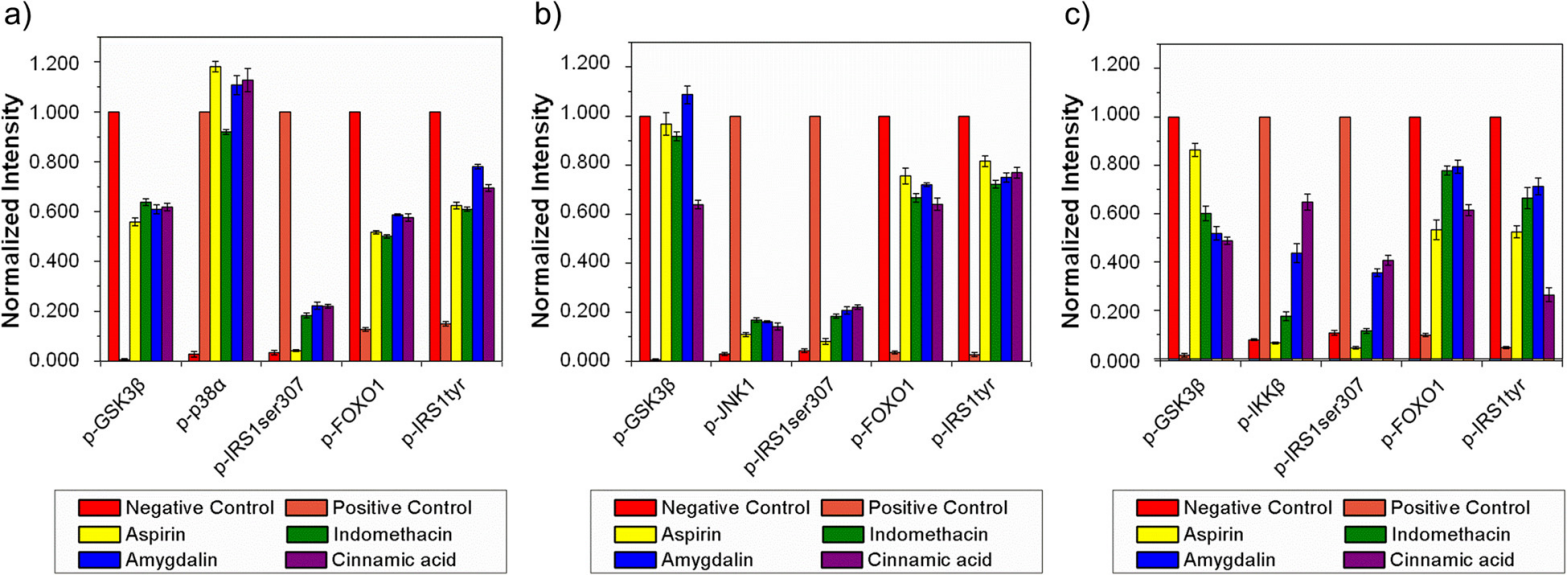
**Quantitative analysis of Figure** 4 **(a,b,c). (a, b, c)**. The fluorescence intensities of the four kinases (p-GSK3β, p-IRS1^ser307^, p-FOXO1, and p-IRS1^tyr^) and each of p38, JNK1, IKKβ, and inhibitor-treated HepG2 cells were normalized to the control, respectively, as shown in Figure 4 **a**, **b**, and **c**. The fluorescence intensities were calculated as described in Methods.

To further elucidate the role of p38α in relation to insulin resistance, we tested insulin signaling kinases, such as GSK3β, FOXO1, IRS1^ser307^, and IRS1^tyr^ in TNF-α-stimulated HepG2 cells pretreated with a 5 μM p38-specific inhibitor SB203580 for 30 min. As shown in Figure 4a, TNF-α-stimulated HepG2 cells show the phosphorylation of p38α along with IRS1^ser307^ in comparison to the negative control group. The SB203580 inhibitor suppressed the activation of p38α and IRS1^ser307^; whereas, the activated phosphor-protein levels of GSK3β, FOXO1, and IRS1^tyr^ were similar to those of the negative control group and had a favorable effect on insulin sensitivity, similar to previous studies using Western blot [15]. To explore the application of the developed assay for pathway studies, we further screened the activities of NSAIDs, such as aspirin and indomethacin, along with two herbal active constituents, such as amygdalin and cinnamic acid. The above four molecules have not been shown to inhibit the p38 inflammatory pathway, but they still inhibited the phosphorylation of IRS1^ser307^ responsible for insulin resistance by acting through other signaling kinases (Figure 4a). We next investigated whether these four molecules can act via the JNK1 pathway. The experimental conditions were similar to those of the p38 screening, except for the use of specific JNK inhibitor SP600125 (4 μM). Positive-controlled HepG2 cells cause activation of IRS1^ser307^ by the JNK1 pathway (Figure 4b). The JNK1-specific inhibitor completely inhibited the phosphorylation of JNK1 and IRS1^ser307^. The four inhibitor molecules inhibited the JNK1 pathway to almost the same extent and enhanced insulin sensitivity, as shown in the restored extent of phosphorylation of GSK3β, FOXO1, and IRS1^tyr^ (Figure 5b). There are several reports of the hypoglycemic effects of naturally-derived active components which have anti-inflammatory activity, such as phenolic acid (cinnamic acid) and cyanogenic glycosides amygdalin. The cellular and molecular mechanism of action of cinnamic acid and amygdalin has not been well elucidated in conditions mimicking inflammation-associated insulin resistance. Although cinnamic acid and amygdalin were expected to act through inhibition of the IKKβ and NF-κB pathway, they acted via the JNK1-dependent pathway to improve insulin sensitivity. Our results are congruent with past studies conducted on mouse liver FL83B cells using phenolic acid, such as cinnamic acid. Cinnamic acid up-regulates the expression of insulin signal associated proteins, including phosphatidylinositol-3 kinase, glycogen synthase, and insulin receptor; it also increases the uptake of glucose, and abates insulin resistance [16]. Our results agree with the JNK1 pathway studies conducted on hepatocytes and mouse liver cells [17]. Finally, we used the specific IKKβ inhibitor (2 μM) to find the relation to the insulin signaling. The IKKβ inhibitor also inhibited IRS1^ser307^ via suppression of IKKβ kinase. Among the four molecules, aspirin substantially inhibited IKKβ kinase activation followed by indomethacin; whereas, amygdalin and cinnamic acid showed moderate inhibition (Figures 4c and 5c). Figure 4e is a Western blot analysis to show suppression of TNF-α-induced insulin resistance induced by the four test molecules. Activations/deactivations of seven different kinases were observed to compare the results obtained with QDot multicolor cellular imaging. Although detection sensitivity of Western blot is poorer than that of QDot multicolor cellular imaging, the results by Western blot were matched with those by QDot multicolor cellular imaging. Figure 5 represents the quantification of cellular images in Figure 4. Our results are in line with past studies supporting the major role of IKKβ in insulin resistance [18].

In insulin resistance-free conditions, insulin phosphorylates Forkhead box-containing O subfamily-1(FOXO1), resulting in the inhibition of transcriptional activity, leading to reduced glucose production. Moreover, insulin phosphorylates GSK-3, thereby leading to an increase in glycogen synthase activity. The glycogen synthesis is controlled by glycogen synthase kinase-3 (GSK-3). On the other hand, TNF-α stimulation inhibits the phosphorylation of FOXO1 kinase and increases glucose production. TNF-α is known to inhibit the activities of many components involved in insulin signaling cascade. Insulin receptor, IRS, and glucose transporter 4 (Glut4) are representative proteins to be inhibited by TNF-α. Particularly, IRS has received great attention as a major target in TNF-α-induced insulin resistance. Insulin signaling is suppressed by an increase of serine phosphorylation of IRS proteins caused by TNF-α. A few mechanisms have been reported to be involved in serine phosphorylation of IRS proteins, such as inhibition of tyrosyl phosphorylation of IRS-1 [19] and proteasome-mediated degradation of IRS-1 [20]. Interaction between insulin receptor and IRS-1 is interfered by the inhibition of tyrosyl phosphorylation of IRS-1; as a result, IRS-mediated insulin signaling is suppressed. Serine phosphorylation of IRS-1 by TNF-α can be achieved through activation of several serine kinases, including kinases related to the inflammatory pathway, such as JNK. The suppression of IRS-mediated insulin signaling leads to reduced GSK-3 and FOXO1 phosphorylation, followed by decreased glycogen synthesis and increased gluconeogenesis. Our results showed that cinnamic acid and amygdalin enhanced the phosphorylation level of GSK-3 and FOXO1. As a result, glycogen synthesis was increased and glucose production was reduced.

## Conclusion

The above results corroborate that QDot multicolor cellular imaging can be applied to investigate the molecular pathogenesis and inhibitor studies of multiple signaling pathways (i.e., insulin signaling and the inflammatory pathway) which share common intracellular proteins. Compared to Western blot, QDot multicolor cellular imaging enabled direct and concurrent accesses to intracellular target proteins related to TNF-α-induced insulin resistance. The designed monitoring proved that cell lysis-free QDot multicolor cellular imaging was a powerful tool for identifying novel molecular targets for suppressing inflammation in insulin resistance. The developed assay successfully verified that cinnamic acid and amygdalin suppressed TNF-α-induced insulin resistance mainly via the inhibition of JNK.

## Methods

### Materials

General chemicals, including a glucose assay kit and TNF-α, were obtained from Sigma-Aldrich (St. Louis, MO, USA) and were of the highest purity available. The glycogen assay kit was purchased from BioVision (Milpitas, CA, USA). The phospho-p38α, phospho-JNK1, phospho-IKKβ, phospho-IRS1^ser307^, phospho-IRS1^tyr^, phospho-GSK3β, and phospho-FOXO1 antibodies were obtained from Santa Cruz Biotechnology (Dallas, TX, USA) and conjugated with 565, 565, 605, 705, 525, and 655 QDots, respectively, using an antibody conjugation kit from Invitrogen (Carlsbad, CA, USA).

### Cell culture

HepG2 cells were obtained from the Korean Cell Line Bank (Seoul, South Korea) and cultured in Dulbecco's modified Eagle's medium supplemented with 10% fetal bovine serum, 100 μg/ml penicillin, and 100 μg/ml streptomycin. All of the cells were cultured in 25 cm^2^ cell culture flasks at 37°C in a humidified atmosphere of 5% CO_2_.

For inhibitor studies, HepG2 cells were plated in serum-free DMEM media for 24 h prior to testing. Inhibitors were added 30 min before stimulation with 10ng/ml TNF-α. After 5-h stimulation with TNF-α, the cells were treated with 100 nM insulin for 10 min.

### Analysis of glycogen content

Glycogen content in various inhibitor-treated HepG2 cells was measured with a glycogen assay kit according to the manufacturer’s instructions.

### Glucose production assay

Cells were washed three times with PBS to remove glucose, incubated for 16 h in 1 ml of glucose production medium (glucose and phenol red-free DMEM, containing gluconeogenic substrates, 20 mm sodium lactate, and 2 mm sodium pyruvate), and in the presence of 1 nm insulin during the last 3 h. 300 μl of the medium was sampled for the measurement of glucose concentration using a glucose assay kit.

### QDot conjugation with specific antibody

The p38α, JNK1, IKKβ, IRS1^ser307^, IRS1^tyr^, GSK3β, and FOXO1 antibodies were conjugated with 565, 565, 565, 605, 705, 525, and 655 QDots, respectively, using an antibody conjugation kit. The used QDots are semiconductor materials that consisted of core, shell, and polymeric coating. The core is made up of cadmium selenide and passivated with zinc sulfide as the shell. The QDot composed of core-shell is highly hydrophobic. In order to enhance water solubility and conjugate efficiently to biomolecules, QDots surfaces were modified with the amine-functionalized polyethylene glycol (PEG) linker. Conjugation steps were carried out according to the manufacturer's instructions. QDots were activated with SMCC used as an amine-to-thiol crosslinker to the maleimide-nanocrystal surface. To break the disulfide bond in antibodies and to expose free sulfhydryls, the antibodies were treated with dithiothreitol (DTT). Size exclusion chromatography was performed to remove excess SMCC and DTT. Maleimide-QDots were incubated with the reduced antibodies for 1 h. By this reaction, antibodies were covalently bound to QDots. The excess maleimide groups were removed by β-mercaptoethanol. A separation media column supplied in the kit was used to eliminate unbounded QDots and antibodies. The maximum emission wavelengths of phospho-GSK3β, phospho-IKKβ, phospho-p38α, phospho-JNK1, phospho-IRS^Ser307^, phospho-FOXO1, and phospho-IRS^tyr^ are 525, 565, 565, 565, 605, 655, and 705 nm, respectively.

### QDot immunoassay using QDot multicolor cellular imaging

HepG2 cells were separately seeded into 12 well culture plates (5 × 10^5^ cells/well). After 24 h, the cells were washed twice with 1× phosphate-buffered saline (PBS), treated with accutase and incubated at 37°C for 10–15 min. The detached cells from the culture plates were centrifuged at 220 *g* for 3 min. After removing the supernatant, the cells were treated with 3.7% formaldehyde for 15 min. The cells were then rinsed twice with 1 × PBS and centrifuged at 220 *g* for 3 min. The cells were treated with 0.2% saponin for 15 min to increase the pore size of the cell membrane, followed by washing twice with 1 × PBS and centrifugation at 220 *g* for 3 min. After the QDot-antibody conjugate, its concentration was determined to be 2 μM using an absorption spectrophotometer. QDot-antibody conjugates (p38α, JNK1, IKKβ, IRS1^ser^, IRS1^tyr^, GSK3β, and FOXO1) were diluted (1/200) in 100 μl of blocking buffer (1% BSA in 1 × PBS). The HepG2 cell pellet was immersed in the diluted QDot-antibody solution of 100 μl and kept for 1 h at room temperature. The cells were then washed twice with 1 × PBS and centrifuged at 220 *g* for 3 min. Intracellular monitoring of the five different kinases were simultaneously carried out at 525 nm, 565 nm, 625 nm, 655 nm, and 705 nm emission wavelengths to observe their phosphorylations induced by tested drugs [21-23]. The fixed cell formed by formaldehyde treatment is a kind of dead cell that maintains its morphology. The fixed dead HepG2 cells were treated with Qdot-antibody probes for the immunostaining. Accordingly, toxicity inherent to Qdots does not affect drug-induced up-regulation or down-regulation of intracellular target proteins. Imaging analysis was performed using commercially available software (MetaMorph, Version 7.1.3.0, Molecular Devices, Sunnyvale, CA, USA).

### Calculation of normalized intensity

Figure 5(a-c) shows the quantification of fluorescence intensities in the cell images obtained with QDot multicolor cellular imaging. Fluorescence intensities of p-GSK3β, p-FOXO1, and p-IRS^tyr^ were normalized according to the following equation:

$$ \mathrm{Normalized}\ \mathrm{Intensity} = \left({\mathrm{I}}_{\mathrm{s}}/{\mathrm{I}}_{\mathrm{n}}\right), $$

The fluorescence intensity of the inhibitor-treated cells is represented by I_s_, and I_n_ represents the fluorescence intensity of cells in the negative control group.

The normalized fluorescent intensities of p-p38, p-JNK, p-IKKβ, and p-IRS^ser307^ were calculated according to the following equation:

$$ \mathrm{Normalized}\ \mathrm{Intensity} = \left({\mathrm{I}}_{\mathrm{s}}/{\mathrm{I}}_{\mathrm{p}}\right), $$

The fluorescence intensity of the inhibitor-treated cells is represented by I_s_, and I_p_ is the fluorescence intensity of cells in the positive control. All of the intensities in Figure 5 were the averaged value of cellular intensities obtained from three different cellular regions. The standard deviation was represented as the error bar in Figure 5.

### Western blotting

HepG2 cells (1 × 10^6^) were lysed using Lysis-M solution (Roche, Manheim, Germany). Electrophoresis was performed on 12% sodium dodecyl sulfate-polyacrylamide gel (SDS-PAGE) by loading 60 μg of protein from HepG2 cells. The proteins were transferred to a polyvinylidene difluoride (PVDF) membrane (BioRad, Hercules, CA, USA) after the electrophoresis. Appropriate primary antibodies were used for Western blot analysis. Membranes were blocked overnight at 4°C in 1 × PBS, containing 0.05% TWEEN® 20 and 5% bovine serum albumin. Each specific antibody at a dilution of 1:1,000 was used in the Western blot analysis. Similarly, beta actin antibody at a dilution of 1:1,000 was used for the detection of beta actin (positive control). Primary antibodies were detected via a secondary peroxidase-conjugated antibody at a dilution of 1:10,000 (Dako Ltd., High Wycombe, Bucks, UK). An enhanced chemiluminescence detection kit (Roche, Basel, Switzerland) was used for signal detection.

## Abbreviations

TNF: Tumor necrosis factor
JNK: c-Jun N-terminal kinase
IKKβ: Ikappaβ kinase
IRS1: Insulin receptor substrate-1
GSK3β: Glycogen synthase kinase 3β
FOXO1: Forkhead box O1
